# Exploring interprofessional education priority settings: Perspectives of Faculty of Health Sciences programme managers

**DOI:** 10.4102/hsag.v31i0.3303

**Published:** 2026-04-09

**Authors:** Mpho J. Morule, Shenuka Singh, Ahmed Bhayat

**Affiliations:** 1 Discipline of Dentistry, School of Health Sciences, University of KwaZulu-Natal, Durban, South Africa; 2 Department of Community Dentistry, Faculty of Health Sciences, University of Pretoria, Pretoria, South Africa

**Keywords:** health sciences training, health sciences curriculum, interprofessional education, interprofessional learning, faculty leaders

## Abstract

**Background:**

Transformational leadership is crucial for the support and sustainability of any interprofessional education (IPE) programme. This is because academic leaders are drivers at a macrosystem level, needed to use their influence to guide change and enable the necessary resources to support IPE.

**Aim:**

To explore programme managers’ perspectives regarding priority settings related to IPE in the undergraduate health sciences training.

**Setting:**

Health Sciences University in Gauteng, South Africa.

**Methods:**

A qualitative, explorative study design was used. Primary data were collected through one-on-one interviews, and secondary data involved a targeted review of curriculum content. A thematic analysis was conducted using the Braun and Clark approach and a criterion checklist for curriculum content analysis.

**Results:**

Interprofessional education seems to be covered broadly in certain schools and departments through the shared modules where students from different professions are taught together, and they work together on community projects. The importance of having committed staff members who are responsive to IPE was also identified as valuable resources for the implementation and success of IPE.

**Conclusion:**

The results showed that IPE is implemented in certain schools or departments. However, challenges such as timetable constraints and a lack of IPE coordination could be improved to promote successful IPE implementation. Participants highlighted the need for administrative support across all schools or departments including a dedicated coordinator and flexible timetable to accommodate IPE-related activities.

**Contribution:**

The study findings provide insights that can help to identify opportunities to strengthen the existing IPE activities and improve the implementation thereof.

## Introduction

Interprofessional education (IPE) is essential in preparing health sciences students for effective collaborative practice in healthcare settings (Katoue et al. 2022). The World Health Organization (WHO) recommends IPE as a viable educational strategy to prepare health students for future interprofessional and collaborative practice (IPCP) (WHO 2011). Since its inception, IPE has received increasing attention, with growing interest in the IPE curriculum and its implementation. An emphasis is that the responsibility of health institutions is not only to produce a health professions workforce that is responsive to healthcare needs but also to ensure that its graduates can practice such knowledge and skills collaboratively (Interprofessional Education Collaborative [IPEC] 2011; Müller & Couper 2021). It is for these reasons that previous studies have criticised the traditional discipline-specific training as it has limitations in facilitating interprofessional communication and collaboration (Belrhiti, Van Belle & Criel 2021; Van Wyk & De Beer 2017).

The development of the IPE curriculum is best planned collaboratively, involving students, educators and academic leaders from the different professions. While the educators’ role is to facilitate teaching and learning experiences, equally so is strong leadership crucial for the support and sustainability of any IPE programme (Black et al. 2022). Evidence has shown that commitment from institutional leadership on all levels is essential to the successful delivery of IPE initiatives (Dallaghan et al. 2016; Shrader et al. 2022; Watts et al. 2020).

Guraya and Barr have argued that support provided at a faculty level by programme managers, for example, through the implementation of educational policies and making resources such as learning materials available, can directly and indirectly influence teachers in their delivery of teaching and learning practices (Guraya & Barr 2018).

This point was emphasised by Bogossian et al., who reported several pre-requisites at a system level for IPE uptake, implementation and sustainability, including strong leadership, structures and processes that facilitate collaboration and impact (Bogossian et al. 2023). Both these studies noted that such institutional support is critical in ensuring access to resources for the development and implementation of IPE. The lack of administrative support has been identified as a barrier to IPE within education, in addition to the perceived limited organisational support for IPE events.

Substantial efforts have been made to integrate IPE initiatives into the education of health students in many institutions in developed countries to prepare students for collaborative practice (Müller & Couper 2021; Van Wyk & De Beer 2017). However, most South African universities are still in the early stages of the IPE movement, some of which have limited implementation of IPE or are facing hindrance. Major barriers hinder IPE implementation, and these include limited financial and human resources, lack of institutional support, variations in the curricula of different programmes, difficulties in setting up common courses and activities for IPE, and limited, lack of student/faculty enthusiasm and interest in IPE and diversity in location and settings of schools (El-Awaisi et al. 2016). As highlighted in the literature, IPE has little chance of success without faculty buy-in; consequently, programme managers are considered key role players in the success and sustainability of IPE at the universities. It was for these reasons that programme managers were recruited to form part of the study.

Previous IPE studies, both locally and internationally primarily used quantitative methods focusing on students, with few reports on faculty or academic staff (Africa et al. 2024; Katoue et al. 2022; Moodley & Singh 2018). Studies reported from the programme managers as academic leaders, who could also be enablers of IPE, are minimal. No published study was found that has been conducted on the status of IPE at the current institution. Therefore, the study aimed to explore the priority settings for IPE in the undergraduate health sciences programmes through the perspectives of programme managers by employing a qualitative method. In addition to the data collected from programme managers, a brief curriculum content review was conducted to locate IPE modules within their respective programmes using a checklist template created by the researchers.

## Methodology

### Research design

A qualitative, exploratory research design was undertaken to explore priority setting for IPE from the health science programme managers and target curriculum/programme content.

### Research setting

The current study was conducted at a health science university in Gauteng, South Africa. The Faculty of Health Sciences (FHSs) at the current university provides undergraduate training in the following three schools divided into departments: School of Medicine for medical doctors and clinical associates; School of Dentistry for dentists and oral hygienists; School of Healthcare Sciences for dieticians, nurses, occupational therapists, physiotherapists and radiographers and various postgraduate qualifications. Each school/department thereof is led by a chair of school and departments led by Head of Departments (HODs). For this study, the researcher visited each school to present the nature of the study to the academic leaders, and based on the scope of the study, HODs and chairs of schools were identified and recruited to participate in the study.

### Population and sampling

The study population consisted of all programme managers who are HODs and chairs of school from the FHSs. A total of eight programme managers formed part of the study. For the recruitment of participants, purposive sampling, specifically criterion technique, was applied.

The use of this sampling technique allowed for the inclusion of participants, who met the pre-determined criterion of being academic leader and manager within the FHSs programmes targeted for the study (Babbie & Mouton 2001).

### Data collection tool

Two methods of data collection were used. Initially, a semi-structured interview with programme managers was conducted. The interview schedule consisted of 10 pre-selected questions focused on the objectives of the study, comprised what is in place in terms of IPE, perceived opportunities and potential barriers to implementation, and at the end of the interview, a question was asked on possible suggestions on how to strengthen and improve IPE in undergraduate training. The questions were developed by the researcher guided by the literature study on IPE (Martin et al. 2021).

### Pilot study

Furthermore, the content of the questions was reviewed by the project supervisors and piloted using two expert qualitative researchers who are familiar with IPE topic. The questions in the interview schedule were refined accordingly.

The second data collection method was targeted curriculum content review. Content of the curriculum was reviewed through data extraction table, which was created with predefined fields to organise and compile data from various sources such as programme brochures and health sciences yearbook, which were freely available on the website. The table provided a structured format for recording key details from each predefined field. This included information about the topics taught in the health science programme, IPE-related concepts in the modular content, year of study, collaboration with other programmes, etc.

### Data collection process

Participants were contacted one on one to provide them with the full description of the study and to invite them to participate. This was followed by an e-mail including a consent form together with an information letter explaining the research purpose and an invitation to the one-on-one interview. The potential participants were given as much time as needed to consider whether they wished to participate, and, in the case of a positive decision, they were asked to give consent for participation, and interview was scheduled at a place and time suitable for them. An online interview was considered for participants that were not comfortable with face-to-face contact.

At the time of the interview, participants were reminded of the purpose of the study, and that their participation was voluntary and reassured that all information obtained would be treated with confidentiality and their responses would be addressed by allocating pseudonyms. All participants granted permission to audio record them as they respond to the questions during the interviews. All interviews were conducted between September 2022 and November 2022, with each interview lasting between 55 min and 70 min long. Audio recordings of the interviews were transcribed verbatim by a professional service provider. All interviews were conducted by the principal researcher, who has no prior relationship with the participants. An independent co-coder was appointed to check transcriptions and ensure precise data translations and interpretations of themes (Neuman 2011).

Following the interviews, the researcher further collected relevant documentation containing any information pertaining to the curriculum of undergraduate training that was available (e.g. programme brochure, yearbook, module guide). A standardised data extraction table was used to extract all data that could be sourced from the readily available documents. A checklist criterion was created to assess and review the content of the curriculum such as individual modules from each health sciences programme targeted for the study, and shared modules with IPE-related concepts were noted. Extracted data included module content, year of study and health science programmes.

Triangulation of data was performed by comparing data from the interviews with that from targeted curriculum content. The purpose of triangulating data was to strengthen research findings and confirm whether evidence from interviews aligns with evidence from the curriculum content review (Almeida 2018).

### Data analysis

The data analysis consisted of transcripts of the interviews and documentation from the targeted curriculum content. The principal researcher reviewed all audio recordings while simultaneously reading the transcripts to ensure the data were complete and accurate. Transcripts were read repeatedly for familiarisation with the data.

This was done individually by the principal researcher and one independent co-coder who is a qualified qualitative data analyst. A thematic analysis of the transcripts content was carried out, using the six-steps method described by Braun and Clarke (2006).

Once the coders had familiarised themselves with the data as well as generated initial codes and observations regarding the data, the two coders convened to compare and discuss the codes and themes identified. Coding was done until no further codes were identified. The investigator triangulation was used to ensure rigorousness within the data analysis process, and the utilisation of a qualitative data analysis software, ATLAS.ti V23 Scientific Software Development GmbH, Berlin, Germany, allowed the researchers to generate new codes as well as alter initial codes. During this process, these codes were continuously modified throughout in which certain codes were disregarded, and several others were merged. All themes and subthemes derived were analysed and compared until saturated. The coders discussed and assessed the outcomes of the coding until consensus was reached. The key themes and subthemes are summarised in Table 1 of the result section. The quotes from interviewees are presented in the text of the ‘Results’ section; participants perspectives are illustrated by the quotes written in italics.

**TABLE 1:** Summary of the themes and subthemes.

Themes	Subthemes
1. Status of IPE in the undergraduate programme	1.1 IPE as shared modules 1.2 IPE in collaborative projects 1.3 IPE in clinical training sites
2. Extent to which IPE is covered	2.1 IPE implementation by year level 2.2 IPE learning activities 2.3 IPE learning competencies
3. Opportunities for IPE	3.1 Resources and support available for IPE 3.2 Receptiveness towards IPE
4. Challenges towards IPE	4.1 Attitude towards IPE 4.2 Time constraints and workload 4.3 Staff ability to adapt/resistance to change 4.4 Administration support 4.5 Timetable issues 4.6 Isolation of medical and dental schools
5. Recommendations	5.1 Administration support 5.2 Importance of staff buy-in for IPE 5.3 Need for IPE training 5.4 Funding for IPE opportunities

Note: Not all themes and subthemes are discussed.

IPE, interprofessional education.

### Content analysis

The collected information from the extracted table was facilitated comparison across health sciences programmes through analysing, synthesising and drawing conclusions about the status of IPE practices in each programme.

The tabulated sections were analysed by labelling the shared modules under the heading status of IPE; the year level and duration as the extent to which IPE is covered and the programmes with IPE-related concepts as opportunities for IPE. The findings are summarised in Table 2 of the ‘Results’ section.

**TABLE 2:** Summary of the basic curriculum overview with shared learning modules and interprofessional education-related component.

Module content	Year level	Duration	Programme involved
Leadership and multidisciplinary teamwork. Healthcare systems and legislation. Determinants of health. Introduction to healthcare models (e.g. community-based care, family-centred care, etc.). Professionalism, ethical principles, management of diversity.	Year 1	Semester 2	P1, P3, P4, P7, P8
Leadership. Principles of project management. Communication principles. Health promotion and education, advocacy and literacy. Counselling for health behaviour change.	Year 2	Semester 1	P1, P3, P4, P7, P8
Leadership in community development. Community needs assessment. Planning and implementation of collaborative community-based interventions. Application of principles of monitoring and evaluation.	Year 3	Semester 1	P1, P3, P4, P7, P8
No IPE-related content/component.			P2, P5, P6

*Source*: University of Pretoria’s Faculty of Sciences Brochure, 2022, viewed 08 December 2021, https://www.up.ac.za/yearbooks/2021/MED-faculty/UD-programmes

IPE, interprofessional education.

### Measures to ensure trustworthiness

To ensure trustworthiness, the four criteria proposed by Lincoln and Guba, namely credibility, dependability, conformability and transferability were applied (Lincoln & Guba 1985). Credibility was enhanced by the prolonged engagement with the participants during data collection. Dependability was enhanced by keeping complete and detailed records of all phases of the research process. To enhance confirmability, an audit trail was maintained that included raw data (audio recordings), interview transcripts, interview guide, evidence of methodological processes and transparent data analysis process. Transferability was enhanced by providing a thick description of the context, study participants and research process, which might be used in the replicated context. Data validation was facilitated by an independent qualitative research analysist to make sure that it was correct. Analyst triangulation (ATLAS.ti v23) ensured that the research findings are supported by the data and are not biased by the researcher’s perspectives. Cross-check of the entire analysis process was conducted by the co-authors to ensure the authenticity of the research findings.

The Consolidated Criteria for Reporting Qualitative Studies checklist tool used to assess and enhance the reporting of qualitative research, including interviews.

This helped to ensure comprehensive and transparent reporting by covering aspects such as the research team, study methods, findings, analysis and interpretations (Buus & Perron 2020). The tool aims to improve the clarity, rigour and trustworthiness of qualitative research reports, making them more credible and appealing. Additionally, methodological triangulation of the data was done to increase the credibility and validity of the results (Joslin & Müller 2016).

### Ethical considerations

Ethical approval was obtained from the institution’s Research Ethics Committee (46/2022). Written informed consent was obtained prior to participation from all participants. The participants’ right to privacy and anonymity was ensured whereby their names were protected and they were given pseudonyms throughout the interview recording and reporting of results. Data obtained were stored in a well-secured laptop, and copies of the dataset in ATLAS.ti software were password-protected and made accessible only to the study investigators, with access control to the professional transcriber and the data analyst.

## Results

### Findings from programme managers

Five key themes and subthemes emerged from the semi-structured interviews with the programme managers. Table 1 provides a summary of the key themes and subthemes. The narrated quotes of the participants were used to support the study’s findings and are presented by direct response with pseudonyms such as PM and number, for example, PM1.

### Theme 1: Status of interprofessional education in the undergraduate programme

#### Subtheme 1.1: Interprofessional education as a shared module across different departments

The subtheme revealed the shared module that is taught across some programmes. The following quotes were shared by participants:

‘We have developed an integrated health learning leadership [*IHL*] module across the three years. So, our students participate in year 1, 2 and 3 in this IHL and the module consist of both theory and community visits.’ (PM8)

However, in other schools/departments, IPE activities are still informal or non-existent. The following quotes transpired: ‘currently there is not much taking place in terms of IPE in our departments’ (PM2; PM5); other participant narrated that, ‘I think mostly it’s informal, it is not formalised but it does happen that students connect with other professions and other professional students in their practical or clinical settings’ (PM6).

#### Subtheme 1.2: Interprofessional education in collaborative projects

Participants recognised that educators implement IPE learning activities such as collaborative projects that facilitate and reinforce the principles of interprofessional collaboration. By implementing IPE learning activities, educators can help students develop the skills and knowledge needed to work collaboratively with other healthcare professionals. The following quote is supportive of this subtheme:

‘The students develop a collaborative project, in year one they get more theory, in year two or three they get practical projects, year two they plan and year 3 they implement, they do it in the surroundings urban or peri-urban communities.’ (PM1)

#### Subtheme 1.3: Interprofessional education in clinical training sites

Clinical and IPE practical experiences are perceived as important for students to better understand the roles that different healthcare professionals play in the delivery of patient care. This could be the opportunity for students to apply what they have learned in IPE-shared modules in real-world clinical settings, allowing them to develop interprofessional collaboration knowledge and skills. Participants stated that:

‘We do have students engaged with students from other professions in the clinical training, typically what would happen is that students will participate in ward rounds, where in those ward rounds there will be medical students, medical doctors, clinical associates and allied health professionals as well.’ (PM6)

### Theme 2: The extent to which interprofessional education is covered

#### Subtheme 2.1: Interprofessional education implementation by year level

Findings revealed that IPE activities are introduced in the first year of training and continue to second and third year of study in certain departments. However, other participants further highlighted that IPE could be consolidated in the final year of study when students have a wider understanding of their individual disciplines.

The following quotes were shared by participants:

‘They start from the first year. I think it works very well that they build on the skills from year one where they learn to work together.’ (PM8)

#### Subtheme 2.2: Interprofessional education learning activities

Participants described the IPE-shared modules as theoretical classes and practical projects that students attend with other students. Students participate from the first year to the third year in the IPE modules, which consists of learning components such as introduction to interprofessionalism, interprofessional communication, ethics and leadership. The IPE learning activities that were mostly reported are delivered through lectures and community-based learning:

‘In the first year, second semester the students become part of IHL, and it focuses more in interprofessional communication, some interprofessional skills like conflict management and problem solving, in the second year they learn to do community needs assessments as an interprofessional team, there is a lot of community health principles they learn. In third year they get a small project like a health care education project at an old age, nursery school or something like that.’ (PM8)

#### Subtheme 2.3: Interprofessional education learning competency

The term ‘competency’ is usually used in the context of describing what the designers of the educational input (activity/initiative) intended for the participants/students to achieve. The findings revealed that participants perceived IPE competencies as important skills for students to learn effective collaborative practice. According to the participants’ perspectives, students need to be competent in teamwork and collaborative skills, learn about the scope of different professions and develop a positive attitude towards others. The following codes are supportive of this subtheme:

*Communication skills:* All participants mentioned communication skills as the most important skills as the students learn to interact as a group and with the communities they work with:

‘“Within IPE, I think you need to teach communication,” which I think is very important. I find all these attitudes that the students have towards each other and towards different disciplines is because they do not understand each other.’ (PM7)

*Teamwork and collaboration*: Almost all participants mentioned that students should be taught to work as a team and learn how to be a team member:

‘Our approach to education in our programme is very much focused on self-directed learning, we would say, there’s EPAs, Entrustable Professional Attributes which would include teamwork, the ability to work with another professional as a team in focusing on what is positive for the patient.’ (PM6)

*Understanding the scope and capabilities of different professions:* The need for students to understand the scope and capabilities of different healthcare professions to make appropriate referrals and work effectively with other professionals affirm the commitment teamwork and collaboration:

‘They must learn to understand each other’s roles, it will strengthen their ability to collaborate and work as a team.’ (PM4)

*Positive attitude towards colleagues from different professions:* The importance of developing a positive attitude towards colleagues from different healthcare professions is essential to contribute to effective collaboration:

‘I think the interpersonal skills are very important, skills like open attitude, attitude of a willingness to learn and work with others, so conflict management skills, the ability to negotiate, to solve a problem in a group or within a team, those are very important skills that they need.’ (PM8)

### Theme 3: Opportunities for interprofessional education

#### Subtheme 3.1: Receptiveness to interprofessional education

Participants highlighted that those students and educators that are participating in IPE are receptive and have demonstrated a positive attitude towards IPE activities. Participants’ perspectives were recorded as:

‘I think they are all receptive. It started just in the School of Healthcare sciences which is the five disciplines, [*Nursing, Physiotherapy, Radiography and Human nutrition, Occupational therapy*] and later we incorporated from the Humanities Faculty the Speech and Audiology students as part of, all disciplines have bought in and they’re participating and excited about it.’ (PM4)

While most participants agreed that the educators are receptive to IPE, they are also aware of some resistance in some programmes. As stated by the participant that:

‘[*S*]ome people are negative, some people want to pull out of it because of the challenges, I think the reception is mixed depending on the module.’ (PM7)

#### Subtheme 3.2: Resources and support available for interprofessional education

Participants emphasised the positive aspect of the current IPE activities that provide an opportunity for health science professionals from different departments to interact with one another. The current interprofessional healthcare leadership (IHL) module and the importance of community engagement are regarded as an effective resource to start working with and suggest they could be incorporated throughout the health sciences curriculum. The following quote encompassed these views:

‘I think there is a movement towards increasing the IPEs and we’ve got the whole curriculum committee that’s busy planning this to try and incorporate things like IPE within the new curriculum, but in general.’ (PM5)

### Theme 4: Implementation challenges

#### Subtheme 4.1: Staff ability to adapt/resistance to change

Participants identified resistance to change as one of the factors that are a challenge to IPE implementation. One participant stated that:

‘The willingness to adapt to IPE because this is another barrier, if people are not willing to adapt to the changes. We become accustomed and very comfortable in doing the way we’ve been doing things for years, so it becomes difficult to change.’ (PM2)

#### Subtheme 4.2: Time constraints and workload

Some participants also noted time constraints and workload as the major challenging factors. One participant commented that:

‘I think the real barriers would be, finding time and place to do this because as you know, our curriculum are already loaded as they are, so there’s got to be a serious shift in how we approach the teaching and the training to identify the need for us to make room and space for IPE because it is going to disrupt the programmes that are put in place, because remember, you are not only looking at your students, you are looking at students across the Schools, to say, “How can I make my timetable to fit in with yours so that the students can all fit in the same venue.”’ (PM2)

#### Subtheme 4.3: Interprofessional education coordination

Some participants indicated that coordinating IPE specifically with different programmes and managing their timetable requires a dedicated coordinator that must take a lead, one response quoted as:

‘And the other barrier would be to coordinate IPE so that the other professionals, or students from the other professions also have time. One thing It’s time on our side and on the other side and coordination of space or venues. Someone must take the lead.’ (PM6)

#### Subtheme 4.4: Administration support

Administrative support is the key facilitating factor for success in IPE; however, some participants felt there was inadequate support received from the FHS, as most of the IPE activities are driven by a single school with few departments involved. One of the quotes recorded as:

‘I do not think the faculty give any kind of support because it is a school driven thing and not a faculty driven thing, and so because it is not a faculty driven thing we see other schools not participating in this for whatever reason.’ (PM1)

#### Subtheme 4.5: Timetable issues

Another major challenge reported by most participants was timetable and scheduling issues. Scheduling is found to be a challenging factor as all departments need to be willing to shift some of their activities to accommodate IPE. Participant highlighted that:

‘Some of the barriers over there is difficulty in merging timetable and making IPE in, as soon as you get two programmes together and then whatever is presented needs to be helpful to both curriculum and that makes a little bit more difficult.’ (PM5)

### Theme 5: Recommendation to improve interprofessional education

#### Subtheme 5.1: Administrative support for interprofessional education

The proposed recommendations addressed the challenges faced with IPE implementation; participants quoted a number of suggestions to improve support for IPE, and comments were noted as:

‘I think we need a module coordinator who is appointed specifically for this IHL because there is quite a bit of work that goes into coordinating, you can imagine the number of students that you deal with in one specific year. I think for me, definitely the administrative support.’ (PM7)

#### Subtheme 5.2: Importance of staff buy-in for effective implementation of interprofessional education

Participants expressed the importance of willingness to actively support IPE for effective implementation, as supported by these quotes:

‘You need staff to buy into it for them to fully invest themselves in that exercise.’ (PM2)

‘So if there is no collaboration among staff members then we are not going to get the full or achieve the ultimate goal of IPE.’ (PM8)

#### Subtheme 5.3: Need for training and workshops to understand the value of interprofessional education

The willingness to improve IPE practice within the institution was acknowledged by the need to conduct workshops to capacitate staff to IPE knowledge:

‘I think it would have to come with some workshop and training so that people can see the value of IPE, and we are not doing because it is fashionable to do it but there are real benefits in doing interprofessional education, So that kind of workshop with the staff is very critical.’ (PM4)

#### Subtheme 5.4: Funding for interprofessional education activities

Participants indicated that the need for funding of IPE activities should be made available. The following statements were made:

‘It would be good if we can have funding, to have a lecturer maybe a module coordinator who focus specifically on IHL and there was a time when we tried to motivate for an appointment like that because we cannot say let’s do IPE and we do not fund it.’ (PM7)

Most participants suggested funding, including PM4 and PM1. This is what one participant mentioned:

‘One resource that might be useful is some transport funds for community engagement, that is one resource that could possibly be a support, I am not aware of other resources. Maybe a grant which is a research Grant which is spread over three years, from that resource we might also find a way of supporting IPE.’ (PM6)

### Findings from curriculum content review

Additional data collected from the targeted curriculum content review are summarised in Table 2, along with the extracted data. The eight targeted programmes for the study are presented with a pseudonym, such as P1. A review of the eight programmes revealed that five programmes from the FHSs have shared modules that are taught collaboratively among these departments. The modules are taught from year one to year three and presented as semester courses, respectively. All these modules are presented in English and have allocated credit bearing in each level of study. Of the eight programmes targeted for the study, the reviewed content showed that three programmes did not have any IPE-related concepts taught throughout their curriculum.

Other aspects of interest include the review of the FHSs brochure where the description of each programme is further described (Faculty Brochure Online 2022). The faculty trains students to work in multidisciplinary teams within tertiary and secondary healthcare facilities. Students gain exposure to primary healthcare in rural and historically disadvantaged regions of South Africa. Clinical training occurs in hospitals and clinics, as well as in communities to equip students with the essential knowledge, professional attitudes and skills to bring hope and an improved quality of life in diverse communities.

## Discussion

This study aimed to explore the programme managers’ perspectives regarding priority settings for IPE in the undergraduate health sciences training. Interprofessional education appeared to be covered broadly, particularly in the school of healthcare science through the module described as IHL where students from different professions are taught together and they work together on community engagement activities.

The findings of this study show that IPE has not only received growing attention at the international level, but significant effort has also been made to implement IPE at the FHSs at this university. This is similar to other universities in South Africa where there also seems to be an intentional implementation of IPE in the teaching curriculum (Joubert et al. 2019; Moodley & Singh 2018; Van Diggele, Roberts & Haq 2021). From the results of this study, IHL is offered as a shared module that teaches students to learn about each other’s professions and the importance of collaboration. Participants stated, and as observed through the targeted curriculum content review, that the module is integrated in nursing, physiotherapy, occupational therapy, dietetics and, although not covered in the scope of the study, radiography programmes.

What could be deduced from the participants and curriculum content review is that the module is introduced as a theoretical part in the first year of study, and educators build on activities into the second and third years with more practical learning implemented at various clinical and community settings. The findings corroborate with results from Van Diggele et al., that the delivery of IPE could differ, and some activity designs are more effective and better suited relating to the learning context (Van Diggele et al. 2021). The early introduction and implementation of a range of settings combined with diversity of instructional methods hold the key for promoting interprofessional learning (Van Diggele et al. 2021).

Some participants argued that it is better to introduce IPE early in the first year while they start with their training and subsequent years in the clinical settings. However, two managers presented different perspectives with conflicting views: one argued that students should first learn their profession before being introduced to IPE with others. In contrast, the opposing view stated that IPE should be introduced in the final year, after students have learned their individual disciplines and when they start working with patients. According to Mohammed et al., the introduction and implementation of IPE can be conventionally assigned to the categories of exposure, immersion and mastery as a continuum of IPE and practice (Mohammed et al. 2021). The exposure phase is primarily preparatory in its organisation and intended for students to come together with students who represent other health professions. Exposure phase helps in positioning the foundation for future partnerships. This phase includes orientation programmes, small group discussions and social activities that promote interprofessionalism. The interactions during exposure will set the stage for the immersion period that requires collaborative interactions that help students focus on learning with, from and about each other’s profession. The immersion phase helps students understand interprofessional values and ethics, along with role clarification, which are essential for effective teamwork and collaboration in healthcare (Mohammed et al. 2021).

The academic year level appears to have different IPE-related topics and competencies expected to be learned by the students such as interprofessionalism, ethics and leadership and conflict management. Most participants perceived the need for students to learn these various skills from the IPE curriculum. Paramount among the skills were communication skills and teamwork as key competencies that must be taught to all students from an early stage. These findings are consistent with study findings by Chitsulo et al., which revealed the need for IPE activities to enhance skills in interprofessional relationships, communication and conflict resolution (Chitsulo, Chirwa & Wilson 2021). The IPEC Core Competencies framework is widely recognised as a foundational model in healthcare education (IPEC 2023). It identifies essential knowledge and skills for healthcare professionals to effectively collaborate and provide patient-centred care. These competencies include values and ethics, roles and responsibilities, interprofessional communication and teamwork (IPEC 2023).

The results of this study showed that, even in different study settings, the reported barriers were similar across the various types of IPE implementation, with the most frequent challenges being logistical constraints, such as scheduling issues, inadequate resources, conflicts with the academic calendars and clinical rotations of different health schools (Oudbier et al. 2024; Van Duin et al. 2022). Van Diggele et al. stated that the key factors for successful implementation of IPE are having a culture that supports IPE and dedicated leadership teams working alongside healthcare teams that embrace IPE (Van Diggele et al. 2021). These include factors relating to individual health professionals, health profession teams and system-related factors. Individual factors identified as contributors to effective IPE include interest and willingness to learn, openness to learn from others, openness to innovation and exposure to interprofessional learning opportunities (Arulappan et al. 2021).

Studies have shown that for IPE uptake to be improved, several pre-requisites were identified as important at the system level, including organisation and team levels and supportive management systems (Ahmady, Mirmoghtadaie & Rasouli 2020; Martin & Sy 2021). In universities where limited organisational support was noted, it usually resulted in problems accessing resources such as time, space and finances for IPE (Soemantri et al. 2019). Similar to the current study, a review by Abu-Rish et al. revealed that scheduling was identified as the most frequently reported barrier to IPE implementation, followed by limitations in faculty and staff time, insufficient funding and inadequate administrative support (Abu-Rish et al. 2012). These studies underscored the need for funding and administrative support and coordinated efforts to plan and implement IPE activities.

Participants in the current study applauded the work achieved by the IPE committee to ensure successful delivery of the IPE programme. Thus, they regarded having such committee as an enabler for IPE implementation.

Participants also perceived the need for training and funding in IPE as important for successful IPE implementation. These findings are consistent with Chitsulo et al., which revealed that participants perceived the need for formal training prior to engaging in IPE to boost confidence and awareness of IPE and its benefits (Chitsulo et al. 2021).

Contrary to other studies, not much was reported on IPE assessments by participants in this study and as observed in the curriculum content review. This was reported in other studies, which highlighted that evaluating the influence of IPE on health sciences student outcomes is a key focus in the sphere of health education evaluations (Busari, Moll & Duits 2017). There are many measuring tools and methods that can be used to quantify students’ knowledge, skills and competencies when participating in IPE initiatives. These measures can include self-assessment surveys, where students can evaluate their readiness to IPE. Additionally, Objective Structured Clinical Examinations such as standardised patient interactions where students’ coordination of various patient care situation and collaboration abilities with different healthcare practitioners are closely evaluated (Abeyaratne, Lim & Krishnan 2024; Norris et al. 2015; Parsell & Bligh 1999). The other measure considered could be peer assessment. Under this framework, colleagues grade each other based on their level of collaboration, professional conduct and communication skills, all demonstrated throughout collaborative learning tasks (Sadikan & Ariffin 2024).

Developing a culture for IPE requires academic staff, students and managers from different disciplines that value IPE and are encouraged to work collaboratively to create a shared perspective and shared objectives. Studies indicate that addressing resistance to IPE requires ongoing support, development and incentives for lecturers involved in IPE activities (Sadikan & Ariffin 2024). Recognising these challenges and the importance of addressing them is the first step towards integrating IPE into science curricula and fostering a collaborative environment among healthcare professionals.

### Strength and limitations

The strength of this study is on the use of two research instrument tools that ensured triangulation of data and validated what transpired from the interviews to the curriculum content review. Similar studies employed a quantitative approach while this study followed on the previous recommendations as to include qualitative studies and target programme managers and not only educators and students.

The results of this study are not representative of all academic health sciences institutions or universities. The limitation of the study is that this is a single-centre analysis. All the participants were from a single institution; therefore, the findings cannot be generalised to other health sciences institutions or universities.

### Recommendations

The schools or departments can leverage on the positive activities implemented from the existing IPE-related modules presented by the school of healthcare sciences and explore different approaches on how to promote IPE effectively. The lack of IPE training and the lack of a dedicated IPE coordinator are worth exploring by the schools or departments collectively to improve IPE practices and ease the burden of workload on the educators. A curriculum with greater emphasis on IPE would empower students to be well equipped with the necessary knowledge and skills needed to work collaboratively with other healthcare professionals. The active involvement of different health science students in the clinical training sites could be an important component that strengthens IPE activities.

## Conclusion

The study revealed that IPE is covered to some extent in the undergraduate health sciences training through the IPE-related modules that are shared across some programmes. This study highlighted the programme manager perceptions about the need to ensure access to resources that should promote IPE activities, such as the appointment of a dedicated IPE coordinator to help with easing the burden of workload on the educators. Participants perceived the need to create more opportunities to support academic staff with funding, among other resources, for IPE training to improve IPE knowledge and promote a culture of IPE practices and collaboration through the support for flexible timetabling. This will help instil an interprofessional culture and break the siloed approach.
